# Supplementary material for: Association between HLA DNA Variants and Long-Term Response to Anti-TNF Drugs in a Spanish Pediatric Inflammatory Bowel Disease Cohort
Source: Int J Mol Sci. 2023 Jan 16;24(2):1797. doi: 10.3390/ijms24021797 (PMC9861004; doi:10.3390/ijms24021797)
Supplement: Supplementary file 1 [file ijms-24-01797-s001.zip › ijms-2106558-supplementary.pdf]

Table S1. Clinical characteristics of pediatric patients classified according to their haplotypes and risk to failure of anti-TNF drug.

Characteristics	Low risk (n=109)	Medium risk (n=133)	High risk (n=98)	*P* value
Sex				
Male, n (%)	69 (33.7%)	79 (38.5%)	57 (27.8%)	0.478
Female, n (%)	40 (29.6%)	54 (40%)	41 (30.4%)	
Age (years)				
At diagnosis, median (IQR, range)	11.2 (4.6, 0.9-16.3)	11.4 (4.4, 2-17.29)	10.7 (3.8, 0.7-17.3)	0.426
At start of treatment, median (IQR, range)	12.1 (4.5, 1.4-17.3)	12.1 (4.4, 3-17.5)	12.3 (3.6, 1.1-17.3)	0.582
Months from diagnosis to initiation of therapy, median (IQR, range)	6.1 (17.6, 0-129.6)	6.1 (11.1, 0-106)	9.8 (16.9, 0-125.8)	0.427
Type of IBD				
CD, n (%)	78 (32.5%)	99 (41.3%)	63 (26.3%)	0.273
UC, n (%)	29 (31.2%)	32 (34.4%)	32 (34.4%)	
IC, n (%)	2 (28.6%)	2 (28.6%)	3 (42.9%)	
First or second biological treatment				
1st, n (%)	105 (33%)	126 (39.6%)	87 (27.4%)	**0.034**
2nd, n (%)	4 (18.2%)	7 (31.8%)	11 (50%)	
Drug				
Infliximab, n (%)	70 (30.7%)	93 (40.8%)	65 (28.5%)	0.768
Adalimumab, n (%)	39 (34.8%)	40 (35.7%)	33 (29.5%)	
Response to anti-TNF				
Yes, n(%)	94 (34.8%)	106 (39.3%)	70 (25.9%)	**0.010**
No, n (%)	15 (21.4%)	27 (38.6%)	28 (40%)	
Months until failure, median (IQR, range) (n=70)	26.6 (37.5, 0.47-103.5)	23 (32.2, 0.46-108)	18.5 (29.8, 0.53-92.4)	0.746
Concomitant immunomodulator (n=330)				
Yes, n (%)	92 (32.2%)	109 (38.1%)	85 (29.7%)	1
No, n (%)	12 (27.3%)	21 (47.7%)	11 (25%)	
Type of immunotherapy (n=283)				
Azathioprine	84 (31.6%)	102 (38.3%)	80 (30.1%)	0.616
Mercaptopurine	1 (33.3%)	1 (33.3%)	1 (33.3%)	
Methotrexate	6 (42.9%)	5 (35.7%)	3 (21.4%)	

Bold, *P* value < 0.05; IQR, Interquartile range; CD, Crohn's disease; UC, Ulcerative colitis; IC, indeterminate colitis.
